# A Structural Analysis of Proteinaceous Nanotube Cavities and Their Applications in Nanotechnology

**DOI:** 10.3390/nano12224080

**Published:** 2022-11-20

**Authors:** Fabian Heide, Jörg Stetefeld

**Affiliations:** Department of Chemistry, University of Manitoba, Winnipeg, MB R3T 2N2, Canada

**Keywords:** protein nanotube, coiled coil assembly, cavity, structural examination, internal physicochemical properties, biocompatible nanomaterial, carrier system, biotechnology

## Abstract

Protein nanotubes offer unique properties to the materials science field that allow them to fulfill various functions in drug delivery, biosensors and energy storage. Protein nanotubes are chemically diverse, modular, biodegradable and nontoxic. Furthermore, although the initial design or repurposing of such nanotubes is highly complex, the field has matured to understand underlying chemical and physical properties to a point where applications are successfully being developed. An important feature of a nanotube is its ability to bind ligands via its internal cavities. As ligands of interest vary in size, shape and chemical properties, cavities have to be able to accommodate very specific features. As such, understanding cavities on a structural level is essential for their effective application. The objective of this review is to present the chemical and physical diversity of protein nanotube cavities and highlight their potential applications in materials science, specifically in biotechnology.

## 1. Introduction

Nanotechnology is quickly evolving, with novel materials being produced on a rapid scale. These nanomaterials range from fibers and sheets to tubular designs [1,2] that are based on various different compositions such as metallic or carbon-based materials [3]. In particular, carbon-based nanotubes, which include single- and multiwall tubes, have found recent applications in many scientific fields such as medicine, energy storage and fuel cells [4]. Adding to these are the advances made with protein-based nanotubes, which offer different properties from those of classical carbon-based nanotubes [5,6]. Although it would seem that biologically based nanotubes offer little room for direct design, current studies have shown that the high complexity of proteins can be an advantage for biotechnological designs that offers a novel perspective on many applications.

Proteins are macromolecules that are naturally expressed by every living organism to serve varying functions. Proteins can vary in length and shape and are generally made up of a set of 20 standard amino acids that arrange into distinct protein folds to allow for high functional diversity. The chemical side chains of these amino acids can consist of functional groups that give proteins their positively or negatively charged regions and allow for the formation of various covalent and noncovalent chemical interactions with other proteins or ligands. The side chains of amino acids can also be nonpolar and are often buried on the inside of the protein folds. Some naturally occurring proteins such as collagen and the HIV-1 envelope protein assume helical secondary structures where multiple helices can come together to form coiled coil assemblies [7]. The formation of these coiled coils often allows for structural cavities that are then termed protein nanotubes. As proteins are highly modular and offer a high chemical diversity due to the many possible combination of amino acids and oligomeric states, the shapes and sizes of the nanotube cavities vary immensely. Recent research has focused on examining the structures of nanotube cavities for potential applications in fields such as drug delivery [8,9], biosensors [10,11] and energy storage [12,13].

The chemical diversity of a proteinaceous nanotube to assume a specific size, shape and function can make original designs extremely complicated, especially if the nanomaterial is to be used for a specific application. Thus, many studies have focused on the repurposing of known natural nanotubes for application development [8,14,15,16]. However, the intelligent design of stable de novo nanotubes with varying structural and functional properties that are intended for specific purposes has also found great interest [17,18,19]. Regardless of the process for engineering these nanotubes, proteins offer unique properties over other nanomaterials such as low toxicity, high biodegradability and high modularity due to their chemical diversity [5]. Although other applications are possible, large strides have been made in the medical field, where protein nanotubes are being used for therapeutic approaches such as drug delivery and protein stabilization. In particular, the cavities of these nanotubes offer safe transport for hydrophobic small molecules that either are harmful or have an inherent low solubility [20,21,22].

Various articles have already focused on the general structures and designs of coiled coils [19,23]. However, due to the rapid advancements in this field, it is necessary to review the structural and functional aspects of nanotube cavities and their ensuing practicality as useful materials. This serves to place protein nanotubes into the context of current nanomaterial developments and features the progress in novel cavity design for a host of applications.

## 2. Naturally Occurring Nanotubes and Their Functions

Protein nanotubes are found across all domains of life and fulfill a wide host of functions ranging from the storage of inorganic molecules in archaea [13] to assisting in the formation of intracellular structural arrangements in eukaryotes [24]. These nanotubes occur in nature as coiled-coil domains that fulfill various roles. Due to the large number of naturally occurring protein nanotubes, it would be impossible to address all their functions. Still, we would like to focus on a few recent findings that increase the understanding of coiled coil properties for the development of novel materials.

Coiled coils are an ancient biomaterial that are present in a large number of archaeal species, some of which form the surface layers of cells. Surface layers evolved to function as a first layer of contact and defense for single-cell organisms, many of which have to endure extreme environmental conditions such as temperatures above 90 °C or pH below 3. As such, the proteins that are present in these surface layers have evolved to be highly stable. For instance, the protein Tetrabrachion from *Staphylothermus marinus* has been shown to be highly resistant to chemical and physical denaturation [25]. Tetrabrachion contains a lipid membrane-anchored stalk region and four protruding arms. A fragment of the stalk was isolated and structurally determined to be a right-handed coiled coil, upon which it was called a RHCC nanotube [26]. The nanotube contains four hydrophobic cavities that can naturally be occupied by octasulfur for the sulfur storage needs of the anaerobic organism. Similarly, the surface-layer protein B (SlaB) in Sulfolobales species has been predicted to form coiled-coil domains [27]. Although protein nanotube fragments have not been isolated, trimeric assemblies that contain central hydrophobic cavities have been identified. In these cases, the coiled-coil domains function as a structural scaffold for the archaeal surface layer. Additionally, the coiled-coil nanotubes function as inherent ligand storage locations over the central cavities.

Coronin A, present in eukaryotic organisms, is an actin-associated protein that assists in the regulation of actin formation [28]. The C-terminal domain of Coronin A forms a four-helix coiled coil, which is necessary for correct actin filament bundling and organization. Although the coiled coil has cavities, no functions have been associated with them, and it is thought that the oligomerization simply serves to assemble and modulate protein complexes. Interestingly though, amino acid mutations in the *a* and *d* positions of the coiled coil allowed for different conformational states that changed the internal cavities’ properties [29]. Studies of Coronin A have demonstrated that coiled-coil domains are more flexible than previously thought as the interhelical noncovalent interactions are highly dynamic [30]. Likewise, the coiled-coil domain of cartilage oligomerization matrix protein (COMP) forms a nanotube that binds multiple different fatty acids. The dynamic nature of the internal cavity accommodates differently sized and shaped molecules, which permits their appropriate internal storage [31]. In both of these cases, the flexible attributes of protein nanotubes allow them to serve their desired function.

Coiled coils are also present in viruses. An example of this is the rotavirus nonstructural protein 4 (NSP4), which functions in viral transcription, morphogenesis and pathogenesis by binding to various host proteins upon infection [32]. The chemical structure assembles into a tetrameric coiled coil with hydrophobic cavities. These cavities naturally bind calcium, which establishes interhelical interactions and stabilizes the nanotube [33]. Studies have shown that the oligomeric assembly of NSP4 is highly dynamic as a single amino acid mutation from a glutamate to a glycine allows for a pentameric assembly at pH 7.5 [32]. These chemical changes occur between different strains of the rotavirus where the dynamic nature of the coiled coil permits varied function under changing physiological conditions.

The natural variety of the coiled-coil motif highlights the possible modularity of protein-based nanomaterials. Although these nanotubes are as ancient as the first archaeal species, they offer many favorable properties such as high stability and flexibility. The various different coiled-coil arrangements have also been shown to serve basic cellular functions that could be advantageous for the development of nanotube materials, specifically when making use of the cavities that exist in many of the coiled-coil assemblies.

## 3. Structural Examination of Nanotube Cavities

**Figure 1:**
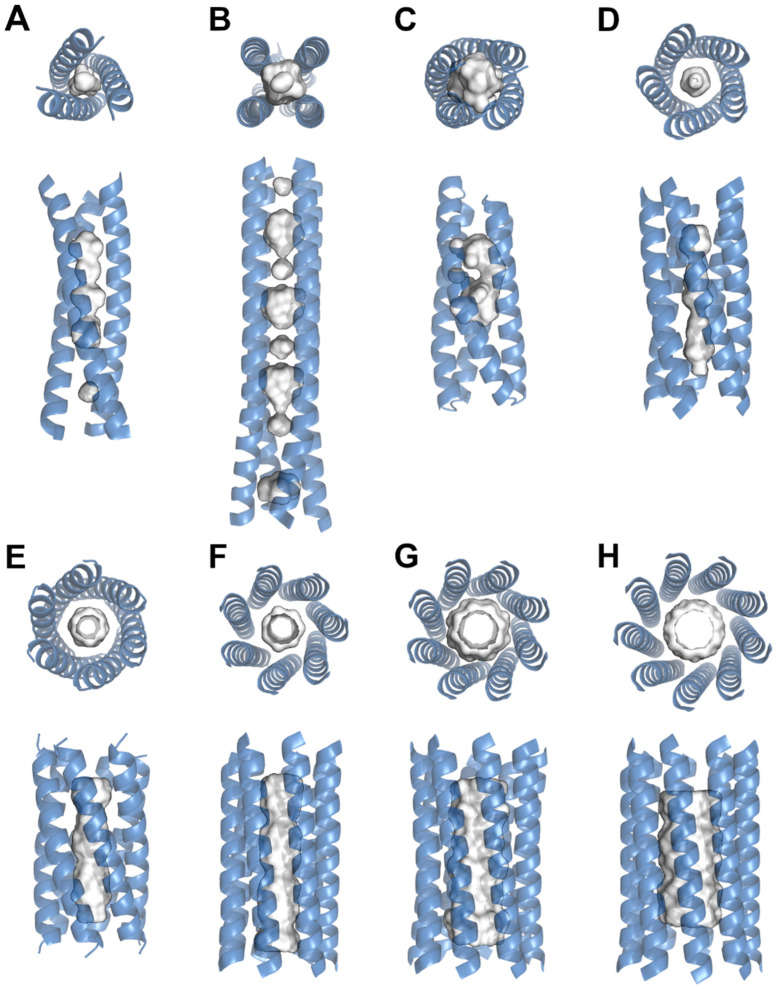
Structural representations of protein coiled-coil nanotubes. The protein coils and cavities for (**A**) Pb(II)C_3_ (PDB: 6MCD), (**B**) RHCC (PDB: 7R6H), (**C**) ABLE (PDB: 6W70), (**D**) CC-Pent (PDB: 4PN8), (**E**) CC-Hex2 (PDB: 4PN9), (**F**) CC-Hept (PDB: 4PNA), (**G**) CC-Type2-II (PDB: 6G67), (**H**) CC-Type2-(GgLaId)4 (PDB: 7BIM) are shown. Cavity sizes and shapes are depended on the oligomeric state and internal residues. Models are based on x-ray diffraction data and were taken from the Protein Data Bank (www.rcsb.org (accessed on 3 October 2022)). Bound ligands were omitted, and the cavity surfaces were generated using the advanced Poisson–Boltzmann solver method based on the solvent accessible surface area with a cut-off of three solvent radii.

Nanotube cavities vary in size, shape and chemical properties (Figure 1). These features depend on the amino acid sequence of the protein and sometimes the chemical environment of the nanotubes [21,34,35,36]. The nonpolar side chains from amino acids such as leucine, isoleucine and valine most commonly form cavities inside of protein nanotubes due to their hydrophobicity and the ‘knobs and holes’ packing as multiple helices come together to form coiled coils [30,37]. The assemblies are often related by symmetric operators that are stabilized by a high number of chemical interactions [38]. The packing of coiled coils has been studied in great detail, which has allowed for the minimal, rational and computational design of novel nanotubes [10,17,19,39,40]. In general, amino acid sequences are designed to follow an *hpphppp* repeat where *h* denotes a hydrophobic and *p* denotes a polar amino acid. The individual positions are also commonly denoted as *abcdef*. This repeat allows for efficient packing where the hydrophobic side chains are buried inside of the nanotube and form interhelical van der Waals interactions [23,41,42]. Meanwhile, the polar residues either interact with the solvent or form additional interhelical noncovalent bonds such as through electrostatic interactions and hydrogen bonds that stabilize the overall structure of the nanotube [43,44,45]. Although this is thought to be the general sequence motif for coiled coils, recent studies have explored different repeat patterns that still assembled into stable coiled coil nanotubes. The many interactions between the helices that assemble a protein nanotube can create an extremely stable structure that resists unfolding at high temperatures or extreme pH [6,9,44]. Even under these varying and extreme environmental conditions, coiled-coil assemblies maintain internal cavities. The preferred packing of a hydrophobic core implies that most internal cavity surfaces are constructed out of nonpolar side chains. A recent systematic analysis of protein cavities showed that smaller cavities tend to be hydrophobic and function to selectively bind ligands through noncovalent interactions [46]. Nevertheless, while hydrophilic cavities tend to be larger in size, the high modularity of proteins allows for a wide diversity in ligand binding that can accommodate a wide range of ligand sizes; ligands can range from simple metals such as calcium [32] and zinc [18] to larger molecules such as capsaicin [14], β-carotene or prodan [10].

Nanotube cavities are generally constructed by the first and fourth (*a* and *d*) positions of a heptad repeat, where higher stability of a coiled-coil assembly is usually achieved by nonpolar residues being buried in the core [39]. These tend to have small side chains with limited flexibility such as those of leucine, isoleucine and valine. However, these trends do not come without exceptions; around 10% of coiled coils have aromatic side chains of phenylalanine, tyrosine and tryptophan in these positions. Despite being extremely bulky and thought to create steric clashes within nanotube cores, aromatic rings form π-π interactions that stabilize the overall structure [47]. The incorporation of these side chains introduces unique chirality on the individual helix structures that have been found to be stable [48]. Additionally, metal binding nanotubes have been shown to include buried polar side chains that in most cases require a bound metal for appropriate stabilization of the nanotube assembly [11]. Due to this, the metal-binding cavities tend to have a very specific volume that tightly coordinates the ligand. Comparatively, hydrophobic cavities vary from spherical to elongated pore-like shapes of varying sizes. These are advantageous for the uptake of nonpolar ligands that sterically fit into the cavity’s volume [10,49]. The appropriate occupancy of cavities has also been shown to improve the stability of nanotubes where the additional interactions allow for better packing of the core [41]. Beyond interactions that occur within the core of the nanotube, other main driving forces for the stabilization of coiled coils are the interhelical interactions along the outer surface [42,43]. Residues in the *e* and *g* positions of the heptad repeat usually consist of polar residues that form salt-bridge interactions on the outer nanotube surface. However, long-range synergistic interactions between the *f*, *b* and *c* positions have also been confirmed [45]. These occur through electrostatic forces that change the hydration of the local environment, where synergistic residues such as glutamate and lysine improve the solvent stability of the nanotube. As such, functional groups on the outer surface determine the overall solubility of the nanostructure and influence the stability of potential cavity spaces.

**Figure 2:**
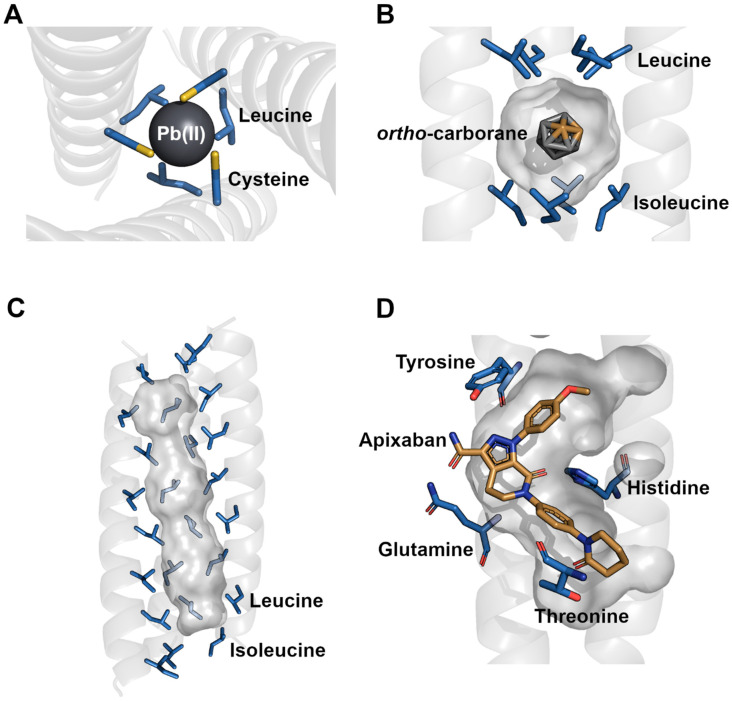
Selected structural models of cavity residues and their interactions with bound ligands. (**A**) The Pb(II)C_3_ (PDB: 6MCD) coiled coil coordinates a Pb(II) atom via cysteine and leucine residues. The coordination is further stabilized by a hydrogen bond network with bound water molecules between the helices. (**B**) RHCC (PDB: 7R6H) in complex with *ortho*-carborane. The nonpolar ligand is stabilized in the hydrophobic cavity by the nonpolar side chains of leucine and isoleucine. (**C**) Cavity space of the CC-Hex2 (PDB: 4PN9) nanotube. The elongated hydrophobic cavity is constructed by alternating leucine and isoleucine side chains which span the entire hexameric nanotube. Three helices were omitted for improved visualization. (**D**) Apixaban binds into the central cavity of ABLE (PDB: 6W70). The polar ligand is stabilized by multiple polar residues including tyrosine, histidine, glutamine and threonine.

Many of these concepts were first identified in naturally occurring nanotubes, and as previously mentioned, coiled-coil motifs occur in all domains of life to serve particular functions through surface interactions or their use of internal cavities. Coiled-coil cavities are often used for ligand storage, for example, the Tetrabrachion cavities of *S. marinus*, which are thought to bind organic sulfur rings for storage as part of the archaeal metabolic pathway [26]. The cavities of the tetrameric nanotube are lined with the nonpolar side chains of leucine and isoleucine, which also partly stabilize the overall nanotube (Figure 2). Two of these cavities have a diameter of 0.8 nm and can bind nonpolar ligands through van der Waals interactions [9,49]. In contrast to the commonly occurring heptad repeat, this coiled-coil assembly is based on an eleven-residue repeat [26]. Free energy calculations have shown that the nanotube is more stable upon ligand binding, which increases the number of nonpolar interactions and thus further improves the packing of the hydrophobic protein core [12]. Interestingly, it is possible for nonpolar ligands to enter the cavities between the helices instead of through the terminal ends of the nanotube [50]. This suggests that in general, protein nanotubes are not completely rigid but, as do most proteins, have dynamic properties that allow for local movement. Helical secondary structures are extremely flexible, which allows for motions such as tilting, bending and unwinding [51]. In larger assemblies, these motions can affect the cavity sizes and shapes, especially since the general packing allows for motion due to the ‘knobs’ being smaller than the ‘holes’ [30].

Structural changes in cavities have recently been documented for Coronin A, an actin-associated nanotube consisting of four helices. The nanotube has an internal cavity that is usually constructed by the side chains of valine and arginine [29]. Varying the amino acids in the *a* and *d* positions changed the internal packing of the nanotube and thus the structural properties of the cavities. The solvent accessible volume compared between the naturally occurring and mutant structures differed from 34.0 to 10.4 Å^3^ [30]. In addition, the asymmetric helical assembly is dynamic, which allows it to twist and change its internal core properties. Even though the cavities do not serve a known function, mutations in the protein sequence change the physicochemical properties of the cavities. Similar structural changes have been demonstrated in the nonstructural protein 4 (NSP4) among rotavirus strains. NSP4 contains a coiled-coil domain with a cavity that is able to bind calcium [33]. The mostly hydrophobic core of the nanotube varies among virus strains, which changes the physicochemical properties of the nanotube [32]. In particular, the polar side chains of glutamine and glutamate that form the calcium binding site are essential for the stabilization of the tetrameric nanotube. Upon changes to these residues, the oligomeric state of the nanotube shifts, which abolishes the cavity and alters the biological function of the nanotube. The structural alterations, particularly in relation to the cavities, that occur in naturally occurring nanotubes help us understand the underlying connections between protein sequences and the resulting structures. This has allowed for the effective design of novel nanotubes with cavities that are used for specific applications.

The scientific findings for naturally occurring nanotubes inspired the pursuit of de novo designs. Many of the concepts that allow helical peptide structures to form nanotube assemblies were translated to the engineering of nanotubes of varying shapes and sizes. Being able to control the physicochemical parameters such as length, number of helices and solubility permits effective application development [6,23,39]. Still, important characteristics of designed nanotubes are the internal packing and the cavity parameters that emerge from the constructed peptide sequence. The proper packing of the nanotube allows for increased stability in the nanomaterial, which is required for applications such as in drug delivery and for biosensors [2,52]. The variables that control nanotube assembly have been documented and reviewed in detail [19,39]. Based on the current knowledge, nanotubes ranging from trimers to nonamers can be designed and produced [23,37]. The different coiled-coil assemblies have varying numbers, sizes and shapes of cavities that can be used for various applications. Similar to naturally occurring nanotubes, the cores of engineered assemblies are generally hydrophobic, which allows for efficient packing and stability. In most cases, the cavities of such assemblies are also lined with hydrophobic side chains of leucine, isoleucine, valine and alanine [46,53]. Variations in the amino acid repeat sequence dictate the arrangements of the helices that influence the physicochemical characteristics of the specific nanomaterial (Table 1). Typical variations in the repeat are aimed at the *a* and *d* positions of the heptad repeat as those are the buried residues that affect the core interactions [11,18,54]. An investigation of all combinations between four different hydrophobic residues in these positions showed that valine and isoleucine allowed for stable nanotube formation with different cavity sizes. While valine in the buried core positions produced a hexameric assembly with a cavity diameter of 4.8–7.7 Å, isoleucine in both buried positions formed an octamer assembly with an increased diameter of 9.0–11.0 Å. Interestingly, phenylalanine or leucine in the *d* position caused nanotube formations in collapsed states with no significant internal cavity. These unique assemblies were induced by the bulkiness of the large side chain of the phenylalanine residue or the high flexibility of the leucine side chain [37].

In addition to the exploration of hydrophobic residues in buried positions, hydrophilic side chains that line cavity surfaces have also been examined. Selective variations of histidine residues in the *a* and *d* positions affected the ability of nanotube cavities to coordinate metals such as Cu(II) and Ni(II) [11]. However, the binding of the metals was required for stable nanotube formation, which was probably due to the otherwise unfavorable packing of internal polar side chains. Still, metal-bound nanotubes formed thermodynamically stable structures with a *T_M_* up to 45 °C. Another study examined changes in cavity-forming residues and their ability to bind Pb(II). Selected amino acids that included combinations of cysteine, leucine and alanine were able to form a hydrogen bond network that was confirmed to bind Pb(II) (Figure 2). This hydrogen bond network included a layer of three interhelical water molecules above the cavity that further stabilized the assembly [18]. In addition to metal-binding cavities, nanotubes that bind larger polar molecules have been characterized. An example of this is the apixaban-binding helical bundle (ABLE) nanotube that was designed to bind apixaban (Figure 2). The cavity between the four protein helices binds a single molecule of apixaban via the four polar side chains of tyrosine, glutamine, histidine and threonine. Although the design of a specific cavity that accommodates the chemical groups of apixaban is highly complex, the nanotube was found to be stable with a *T_M_* of above 95 °C [56]. The high stability and successful complex assembly are an interesting finding since the inclusion of polar residues inside of a relatively small nanotube core would be thought to destabilize any potential coiled coil assembly. In comparison with this interaction and the cavity size, ligands such as lycopene [15] and capsaicin [14] have recently been shown to bind into the extremely large cavities of α-lactalbumin nanotubes. The 200–1000 nm long nanotubes with a diameter of 20 nm were demonstrated to be hollow tubes via transmission electron microscopy with an overall negative charge. Although the chemical interactions were not examined structurally, both otherwise poorly soluble ligands were found to be retained by the nanotube, which stabilized them in solution for up to 28 days [15] and under gastrointestinal conditions [14,57].

These studies emphasize that internal cavities of nanotubes are not limited to nonpolar ligand interactions but can be designed to bind ligands of specific interest that enhance the stability of the entire construct. In general, nanotube cavities increase in size with an increase in the nanotube’s length and the number of helices in the coiled-coil assembly. Due to the many possible side chains that can be engineered into nanotubes, the physicochemical characteristics can also change drastically. This opens up many possibilities for prospective designs of ligand-binding cavities.

## 4. Nanotechnology Advancements in the Design of Nanotubes and Their Cavities

Nanotechnology in protein science has improved drastically as we have come to understand underlying principles such as protein-folding determinants based on sequences and solvents. These concepts were largely supported by early structural and biophysical studies of protein species. In addition, many theories of protein helical structures are analogous to the driving forces of DNA double helix formation [58,59]. Furthermore, although many studies of novel nanotube engineering have aimed to demonstrate our general understanding of helical assemblies, they have inherently pushed the field of protein nanotube technology forward. Based on the properties of proteins, large assemblies can be constructed by short peptides that self-assemble into circular [60,61] and helical [62,63,64] structures. The self-assembly is often driven by the hydrophobic effect, which aims to minimize unfavorable water molecule contacts, creating intermolecular interactions between individual protein units [39]. These interactions are illustrated by two separate nanotube assemblies of synthetic peptides that were designed based on tandem repeats of naturally occurring proteins; while the side-to-side interactions were driven by hydrophobic interactions, peptide ring stacking was stabilized by electrostatic interactions of amino acid side chains. This resulted in nanotubes that had a diameter of around 9 nm and varied in length between 50 nm and 5 µm [63]. The forces that drive assembly formation on the large scale are the same as for smaller coiled-coil assemblies. In both cases, individual protein units consisting of various structure motifs come together to form complex arrangements. In short, the collective knowledge of helical folding and interhelical interactions that form coiled-coil nanotubes has matured the field into the de novo design of functional nanotubes. For this, de novo designs of coiled coils have advanced to the point where it is possible to create algorithmic systems. This is feasible due to the high modularity of protein helices from which functional systems that respond to outside stimuli can be constructed. This was recently featured by the design of a nanotube system that used Boolean functions to gate binding specificity [65]. Protein nanotubes have proven to be ideal for functionally complex systems as oligomeric assemblies have an increased modularity due to consisting of multiple helices. The high modularity and ability to control coiled-coil assemblies have also benefitted the design of novel cavities.

Recent advancements in nanotube stability and cavity formation have largely been based on computational methods that allow for the successful construction of nanotubes. These have allowed for the effective and balanced integration of basic underlying concepts that constrain nanotube formation such as individual helical folding, interhelical forces for assembly and solubility parameters [40,56,66,67]. Guided by additional Watson–Crick base-pairing principles, which also consider hydrogen bond networks within nanotube designs, 101 coiled coils were recently designed computationally. Of these, 63 were successfully produced and isolated and displayed effective hydrophobic core packing that yielded high stability assemblies. However, the incorporation of polar contacts within the core was extremely difficult as large hydrogen bond networks had to be satisfied [44]. Buried polar functional groups commonly introduce unfavourable intrinsic electrostatic forces and steric clashes that reduce stability or even disallow coiled-coil formation. Still, these can be overcome by designing a nanotube that binds a target ligand. An example of this is the de novo design of ABLE, which binds apixaban over the polar side chains of tyrosine, glutamine, histidine and threonine (Figure 2) [56]. Interestingly, the design of this nanotube with an incorporated cavity followed a novel strategy; priority was given to building a ligand-specific cavity, after which the surrounding protein scaffold was built. The eventual satisfaction of intrinsic protein forces generated a stable protein structure. The similar ligand-based construction of a nanotube was achieved by the computationally generated coiled coil of porphyrin-binding sequence 1 (PS1). The nanotube was successfully produced and had improved core stability upon porphyrin binding, which changed the rotamer alignments of two leucine and a tryptophan side chains [66]. The successful production of the nanotube was in part due to considerations of direct nanotube–ligand contacts and distant interactions that stabilize general structure assembly. These concepts can theoretically be applied to ligands of interest where a carrier system is needed to carry out an application.

Aside from the chemical environment that is necessary for ligand binding, the available space also has to be considered. As variations in cavity size are highly dependent on the oligomeric state of a coiled coil, for instance four versus nine helices, some studies have focused on sequence principles that determine oligomeric states [23]. Being able to control the number of helices has the advantage that cavity volumes can be precisely engineered to accommodate ligands of specific sizes. The general trend is that with an increase in the number of helices, the central space increases in volume. This theory was demonstrated by the design of novel α-helical barrels with pentameric, hexameric and heptameric assemblies that had upper limit diameters of 7.4, 7.7 and 10.1 Å, respectively. The nanotubes’ capacities to bind various hydrophobic ligands of different sizes such as 1,6-diphenylhexatriene, prodan and β-carotene were then examined [10]. Interestingly, these structures varied from the usual heptad repeat and followed an *hpphhph* repeat pattern with similar sequences. This resulted in an increased central cavity space that was beneficial for ligand binding. Nevertheless, larger ligands such as prodan only fit into the hexameric and heptameric nanotubes. A similar study demonstrated that raising the oligomeric state of nanotubes can be accomplished by enhancing the flexibility of the *g* position in the *hpphhph* repeat. The presence of a threonine residue resulted in a pentameric coiled coil, while replacing it with a glycine residue, which raises conformational flexibility, resulted in nonamer formation [23].

Based on our current understanding, nonpolar ligands are mainly restricted by the sheer size and shape of the occupying cavity [10,49], assuming that the nanotube core is mainly hydrophobic. Meanwhile, the incorporation of polar ligands into a nanotube has the added challenge of designing an appropriate chemical environment that permits stable core packing [18,68]. These concepts have proven to be valuable in understanding and generating novel nanomaterials that bind ligands for application development. As available internal space is crucial for adequate ligand binding, novel nanotube designs need to consider cavity spaces in addition to sequence components for internal chemical interactions.

## 5. Applications for Nanotube Cavities

**Figure 3:**
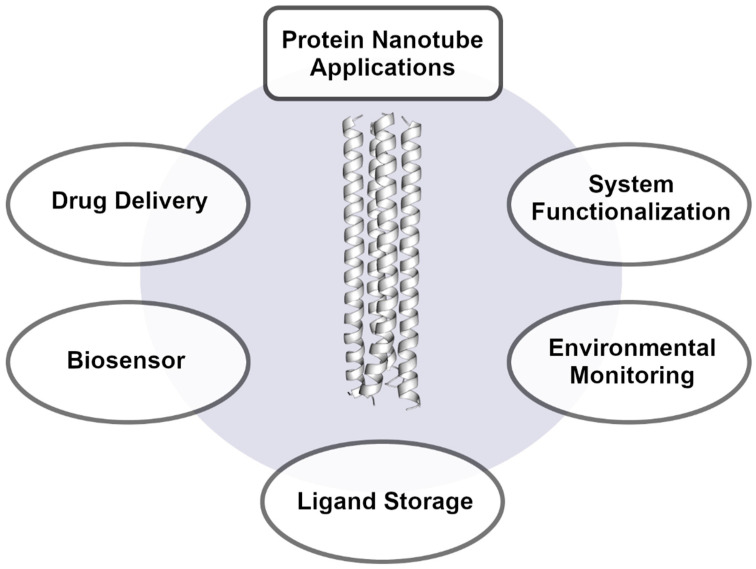
General applications for protein nanotubes in current use or development. These include but are not limited to drug delivery, biosensor, general ligand storage, environmental monitoring and system functionalization applications.

Even though protein nanotubes are considered a niche material in nanotechnology, they offer unique advantages over common materials such as carbon nanotubes. Carbon nanotubes have been explored for multiple applications including energy storage [1], transistors, sensors [69,70] and drug delivery [71,72]. However, their immediate toxicity and unknown long-term health effects make them mostly unsuitable for biological systems [73,74]. Adding to that are the obstacles of commercial production in high yields and of adequate purity [4]. Protein nanotubes can address these issues and prove to be promising nanomaterials, especially for biotechnological applications (Figure 3). In general, organic polymer-based nanomaterials serve as unique carrier systems that can be engineered to bind ligands of interest and transport them to specific target locations for potential release. Due to their organic composition, they offer unique chemical interactions with biological systems and drugs alike [75]. Protein-based nanomaterials exhibit low direct toxicity [8,76], are biodegradable [77] and are highly stable if designed correctly. Although these are preferred characteristics for efficient drug delivery, issues due to cytotoxic effects have been reported [6,8,78] and can occur readily if the drug carrier nanomaterial interacts with nontarget biological systems. Still, cytotoxic effects are being considered in biotechnological developments, and multiple protein nanoparticle–drug complexes have already been approved for therapeutic use [79,80]. The chemical diversity of the various possible amino acids allows for complex engineering to fulfill various functions. These functions include not only the cavity spaces that can be filled with target ligands but also the possibility of functionalizing the outer nanotube surface to associate with molecular targets. Hence, many recent applications have aimed to develop nanotubes into drug carrier systems that attach to cellular targets.

The repurposing and de novo design of novel nanotubes for certain functions has progressed quickly due to the collective increase in coiled coil and cavity knowledge. The naturally occurring protein nanotube RHCC was found to bind a host of medicinal compelling ligands including cisplatin [81] and ortho-carborane [9]. Both of these drugs are applicable towards cancer treatments but lack adequate solubility. In addition, cisplatin is a highly toxic compound [82]. Upon uptake of the drugs into the central hydrophobic cavities, issues with solubility and toxicity are overcome. The nanotube is highly soluble and can be used for drug delivery; assays confirmed that the nanotube enters cellular targets, and additional mouse studies showed that cytotoxic effects of the isolated nanotube were negligible [8]. Similarly, α-lactalbumin nanotubes have been shown to take up various ligands into their large cavities including capsaicin [14] and lycopene [15]. Whereas capsaicin has antimicrobial and pain relieving properties for medical applications, it causes irritation on mucosal surfaces and is poorly soluble in water, which limits the use of the drug [83]. Comparatively, lycopene is a compelling antioxidant that is also restricted by poor solubility and stability [84]. The uptake of these ligands into protein nanotubes was shown to circumvent solubility issues and improve the general stability of the molecules. In the case of the nanotube–capsaicin complex, effective mucus penetration and retention in gastrointestinal tracts of mice was demonstrated [14]. In short, both of these nanotubes show great potential as repurposed drug delivery nanomaterials.

The intelligent design of peptide sequences to create coiled-coil assemblies that bind ligands for drug delivery has also seen large improvements. Initial studies such as the constructions of tetrameric coiled coils experimented with *a* and *d* position substitutions in the heptad repeat to change the physicochemical properties of a central cavity. Various combinations allowed for the uptake of various organic ligands such as adamantane, camphor and corresponding derivatives [17]. The nanomaterial in complex with ligands was shown to be stable, and although subsequent drug delivery was not explored, an early basis for protein nanotube ligand binding was presented. More recent studies have investigated the construction of protein nanotubes around more complex ligands including porphyrin [66] and apixaban [56]. The de novo designs of both nanotubes were directed towards creating a functional ligand-binding protein, which inevitably created intricate cavities. Being able to create nanotubes with binding cavities for target ligands highlights the beneficial modularity of proteins for prospect drug delivery, especially for ligands with difficult pharmacokinetic properties. In contrast, it is also possible to design unspecific cavities that bind a variety of nonpolar molecules. These include novel constructs from a series of coiled-coil nanotubes ranging from trimeric to nonameric assemblies [10,23]. As the number of helices increases, the central cavity space increases in volume, which allowed for the uptake of large nonpolar ligands. In general, nonpolar target molecules show poor pharmacokinetics as they are poorly soluble, resulting in low bioavailability; in certain cases, nonpolar molecules exhibit inherent toxic properties [85,86]. These can easily be overcome by uptake into an appropriately sized, hydrophobic cavity. Still, it should be mentioned that translation from initial nanotube development and the characterization of protein–ligand complexes to a clinical setting has been lacking. This is most likely due to a current emphasis on understanding coiled-coil arrangements and their underlying characteristics. Additionally, although the successful design of novel nanotubes that bind relevant drugs demonstrates our basic understanding, subsequent studies should aim to further establish functional nanotubes as targeted drug-delivery agents.

Even though drug delivery appears to be a prevalent target application for protein nanotubes, other studies have examined potential uses as biosensors. Here, the cavities are filled with ligands that can be detected by absorbance, fluorescence [10], magnetic [68] or even electronic [87] measurements. The essential mechanisms for application development are highly similar to drug-delivery approaches. In this case, however, the presence of ligands is detected using external methods. The central cavities for the nanotube biosensors cover a broad spectrum with varying shapes, sizes and hydrophobicity indices. Upon the uptake of a luminescent ruthenium(II) polypyridyl species into a protein nanocage cavity, the complexes could be detected by spectroscopic methods in cell imaging analyses [21]. Although this complex was not a nanotube material, the cavity was highly reminiscent of hydrophobic cavities in nanotubes where nonpolar side chains stabilize the internalized ligand. Comparatively, nonpolar dyes have been shown to bind Into nanotube cavities nonspecifically, including 1,6-diphenylhexatriene, prodan and β-carotene [10]. These ligands display unique absorbance or fluorescence characteristics that can be taken advantage of for biosensors and diagnostic tools upon targeted delivery by the encapsulating nanotube. Additional imaging applications are possible through the uptake of suitable magnetic resonant compounds. Lanthanides are paramagnetic elements for which a nanotube cavity was designed that coordinates storage with high stability. Multiple Ln^3+^ atoms such as terbium, cerium, neodymium and europium can be stabilized inside the core by the polar side chains of aspartic acid residues and interhelical water molecules that form a close hydrogen bonding network [68]. These protein–ligand complexes have the potential for use as magnetic resonance imaging (MRI) contrast agents. In general, the lanthanide elements are moderately to highly toxic, which leads to serious side effects upon drug usage [88]. However, these can be circumvented with the use of drug-carrier systems. The recent binding studies of functional metalloprotein complexes show that proteins can be used to effectively stabilize metals of interest over various polar residues. In light of this, other metals have also been placed inside of protein nanotubes and could provide other attractive functions such as centers for catalytic reactions. Copper, nickel, zinc and also lead have been shown to bind into the cavities of de novo protein nanotubes [11,18]. These nanomaterials were designed to coordinate multiple metal atoms, which effectively increases the load capacity per nanotube. These studies not only showed that the intelligent design of metal binding nanotubes is possible but also laid the groundwork for the design of soluble catalytic site nanomaterials.

A somewhat unique application of a protein nanotube is the environmental monitoring of toxic polycyclic aromatic hydrocarbons upon crude oil spillage. A nanotube was able to take up and store these nonpolar compounds up to a specific size limit [49], after which it was used as a suspended medium to detect concentrations in an experimental setting. For these experiments, the protein nanotube medium was placed into lake mesocosms for up to 14 days and showed no signs of degradation by environmental factors [89]. As previously discussed, an appropriately designed nanotube provides a highly stable material for versatile functionality. The nonspecific uptake of nonpolar ligands is an appealing concept, as large enough protein nanotubes can also be used to functionalize carbon nanotubes. Numerous proteins have already been used to coat carbon nanotubes to add the advantageous properties of proteins to carbon nanotube applications [16,77]. Common goals for carbon nanotube functionalization are an increase in solubility [90], a decrease in toxicity [91] and options for biological targeting [92]. A recent functionalization study showed that a heptameric protein nanotube was able to retain a single-walled carbon nanotube in its central channel that effectively solubilized the carbon nanotube [93]. This study created a novel nanomaterial that combined the internal properties of single-walled carbon nanotubes with the outer surface properties of proteins.

The strength of protein-based materials is their high diversity and modularity due to the many different sequences that can be constructed. The modularity of proteins also allows for the introduction of chemical labels and modifications to outer protein surfaces that can bind specific biological targets or aid in visualization [94,95]. These chemical labels can be as simple as attaching folate ligands via click chemistry, which enhances cancer cell uptake due to the common overexpression of folate receptors [96,97,98]. Outer surfaces of nanotubes have also been shown to include DNA-binding motifs that can be used for targeting [24,99]. Other targeting approaches include more complex methods such as antibody conjugation [100,101] and the construction of larger nanobodies with high load capacities for potential drug delivery [102]. These allow for improved accumulation around target cells. In addition to biological targeting labels, visualization tags such as fluorescent dyes can be covalently and site-specifically linked to protein surfaces [95,103]. Some investigators have employed noncanonical amino acids that contain reactive side chains and expand on the base number of amino acids, which then allowed for site specific labelling [104,105]. Still, the surfaces of protein nanotubes contain functional groups including primary amines and carboxyl groups that are ideal for quick and less-specific chemical modifications. The quick labeling of amino side chains also allows for the crosslinking of coiled coils that can then form an extended protein material. This has recently been shown where a trimeric coiled coil was linked via covalent chemical crosslinkers and electrostatic interactions to form a larger three-dimensional assembly [106]. In short, the general functionalization of proteinaceous nanotubes is straightforward and usually yields stable nanomaterials.

Nevertheless, the applications for protein-based nanotubes are mostly in the developmental stages. This is in part due to the niche nature of these nanomaterials and current issues with the cost-effective production of sufficient quantities. Additionally, laboratory-to-clinical translation for medical purposes is still in its early stages, with few therapeutic applications in clinical development. Although these can potentially be overcome as technologies advance, current limitations prevent the broader expansion of proteins as nanomaterials. However, a collection of nanotubes have progressed past proof-of-concept studies and show great potential across various fields. In particular, protein nanotubes tend to be focused on in biotechnological settings where carbon nanotubes are generally more difficult to employ. The biocompatibility of proteins combined with their modularity and high stability ratify them as suitable carrier systems. If combined with ligand binding cavities, protein nanotubes present themselves as a promising tool that can be directed towards diverse objectives.

## 6. Conclusions

Our comprehension of protein cavities has advanced to a point where it is possible to design nanotube structures that perform certain functions. Especially in recent years, the development of de novo and repurposed protein nanotubes has generated novel nanomaterials with applications for various technologies. This has in large part been accomplished by understanding the structural basis of internal coiled-coil interactions and factors that drive cavity formation. The cavities in these nanotubes vary in shape, size and chemical complexity, which allows for binding a wide range of ligands. Recent scientific advances have generated complex cavities that bind metals, dyes and drugs that adapt protein nanotubes into functional biomaterials. However, the continued translation of protein nanotubes from developmental stages to real-world applications is imperative for biotechnological progress; protein-based nanomaterials, specifically with ligand-binding cavities, provide unique properties that should be taken advantage of in the right settings.

## Figures and Tables

**Table 1:** Physicochemical properties of selected protein nanotube structures and their cavities.

Protein	PDB ID	Design	Number of Helices	Length (Å)	Width Diameter (Å)	Number of Cavities	Solvent-Accessible Volume (Å^3^)
Pb(II)C_3_ denovo	6MCD	de novo	3	55.2	24.9	1	12.3
Peptide HC02	7P3H	de novo	3	58.9	27.4	1	9.8
alpha4tbA6	4G4L	de novo	4	39.8	29.6	1	76.4
PS1	5TGW	de novo	4	45.1	28.4	1	215
Coronin CC	6AH6	natural	4	91.3	27.4	1	34
Coronin CC double mutant	6ADZ	mutant	4	86.4	27.6	1	10.4
TubY-ctd	7C7Y	natural	4	102.3	30.9	1	74.6
ABLE	6W70	de novo	4	51.1	26.0	1	181.5
RHCC	7R6H	natural	4	78.9	27.0	4	84.8, 78.4, 57.1, 55.4
COMPcc	3V2N	natural	5	72.0	30.4	2	166.4, 143.9
CC-Pent	4PN8	de novo	5	44.7	30.4	1	76.5
CC-Hex2	4PN9	de novo	6	48.6	32.7	1	393
CC-Hept	4PNA	de novo	7	48.8	31.8	1	619.3
CC-Type2-II	6G67	de novo	8	48.7	32.3	1	1638.1
CC-Type2-(GgLaId)4	7BIM	de novo	9	44.8	38.2	1	2178.5

Models were taken from the Protein Data Bank (www.rcsb.org (accessed on 3 October 2022)). Nanotube dimensions were determined based on their solved molecular structure. Bound ligands were omitted and the cavity surfaces were generated using the Advanced Poisson-Boltzmann Solver method based on the solvent accessible surface area with a cut-off of 3 solvent radii. The solvent-accessible volumes were then calculated using CASTp 3.0 [55].

## Data Availability

All the generated data are available in the published manuscript.
